# Overexpression of UNC5B in bladder cancer cells inhibits proliferation and reduces the volume of transplantation tumors in nude mice

**DOI:** 10.1186/s12885-016-2922-9

**Published:** 2016-11-15

**Authors:** Chuize Kong, Bo Zhan, Chiyuan Piao, Zhe Zhang, Yuyan Zhu, Qingchang Li

**Affiliations:** 1 Department of Urology, The First Hospital of China Medical University, 155 Nanjing North Street, Heping District, Shenyang City, Liaoning Province 110001 People’s Republic of China; 2 Department of Pathology, The First Hospital of China Medical University, 155 Nanjing North Street, Heping District, Shenyang City, Liaoning Province 110001 People’s Republic of China

**Keywords:** UNC5B, Bladder cancer, Cell cycle, Migration

## Abstract

**Background:**

The netrin-1 receptor UNC5B plays vital roles in angiogenesis, inflammation, embryonic development and carcinogenesis. However, the functional significance of UNC5B overexpression in bladder cancer remains unclear. In this study, we investigated the role of UNC5B in bladder cancer in vitro and in vivo.

**Methods:**

Stable transfection of the human bladder cancer cell line 5637 with UNC5B (5637-U) was confirmed by real-time RT-PCR, western blot and immunofluorescence assays. UNC5B expression in 5637 and 5637-U cells and mice tumor specimens derived from these cell lines was analyzed by immunohistochemistryand western blotting. Changes in the levels of cell cycle proteins were evaluated by western blotting. Flow cytometry, CCK-8 and scratch tests were used to examine cell cycle distribution, proliferation and migration, respectively.

**Results:**

UNC5B overexpression in 5637 cells inhibited cell multiplication and migration and induced cell cycle arrest at the G2/M phase, meanwhile exhibited changes in the expression of cell cycle-associated proteins, showing that UNC5B may inhibit metastatic behaviors in bladder cancer cells. In addition, tumors generated from 5637-U cells were smaller than tumors generated from control 5637 cells.

**Conclusions:**

Our findings suggest that UNC5B is a potential anti-neoplastic target in bladder cancer progression.

## Background

Bladder cancer is the commonest malignant tumor in men worldwide and is associated with poor prognosis. Despite recent improvements in bladder cancer therapies, mortality rates have remained constant [1]. The therapeutic potential of axon guidance factors and their corresponding receptors in cancer therapy has recently emerged. Among the three recently identified members of the netrin family of axon guidance factors, netrin-1, netrin-3 and netrin-4 [2–5], netrin-1 has received the most attention. Netrin-1 is a 60–80 kD laminin-like protein implicated in promoting cell invasion and angiogenesis and inhibiting apoptosis in glioblastoma, lung cancer and breast cancer [6–8]. Receptors of netrin-1 include DCC, the UNC5H family of proteins (UNC5A, UNC5B, UNC5C, and UNC5D) and neogenin [9]. In-depth studies of UNC5B in tumors have revealed that UNC5B is down-regulated in bladder cancer tissues and that lower UNC5B expression is an independent determinants for recurrence of bladder cancer [10]. These findings suggest that UNC5B may function as a tumor suppressor in bladder cancer. In 2007, Bruno Larrivée et al. demonstrated that UNC5B is down-regulated in the existing vasculature of adults but is re-expressed during angiogenesis and tumorigenesis, indicating that UNC5B is a potential anti-angiogenic therapeutic target [11]. Up-regulated expression of netrin-1 and UNC5B has been observed in breast cancer patients with distant metastasis [8]. In this study, we evaluated the effect of UNC5B overexpression on cell proliferation and migration and the effect of UNC5B in tumors implanted in nude mice. We observed a significant decrease in proliferative and migratory activities after UNC5B transfection, and the size of masses under limbs was reduced in nude mice injected with UNC5B-expressing cells.

## Methods

### Cells, plasmid and transfection procedures

The grade II human urinary bladder cancer cell line 5637 was selected for the UNC5B transfection and nude mouse tumor transplantation experiment because 5637 is a suitable transfection host and has tumorigenic ability. The cells were maintained in RPMI-1640 (Lonza, Verviers, Belgium) supplemented with 10 % fetal bovine serum (FBS) (EuroClone, West York, United Kingdom) at 37 °C in a 5 % CO_2_ humidified incubator. The human recombinant pcDNA-UNC5B-green fluorescent protein (GFP) construct was purchased from GenePharma (Shanghai, GenePharma Co., Ltd). 5637 cells stably expressing pcDNA-UNC5B-GFP (hereafter referred to as 5637-U cells) overexpressed UNC5B. For transfection, 2.5 μg of pcDNA-UNC5B-GFP and 12 μl of Lipofectamine™ 2000 (Invitrogen, Carlsbad, CA) were added to 2 ml of serum-free transfection medium and incubated for 24 h. The transfected 5637 cells were cultured in medium supplemented with G418 (500 μg/ml) (Invitrogen) to select cells stably transfected with pcDNA-UNC5B-GFP for approximately 14 days. Next, the 5637-U cells were cultured in RPMI-1640 medium supplemented with 10 % FBS containing G418 (500 μg/ml). Positive clones were determined by GFP immunofluorescence using a fluorescence microscope (Olympus, Tokyo, Japan). Non-transfected cells were used as controls.

### Real-time RT-PCR analysis

TRIZOL reagent (Invitrogen, Carlsbad, CA) was used for RNA extraction according to the manufacturer’s instructions, and the RNA was quantified using a Thermo Scientific NanoDrop ND-100 (Wilmington, DE, USA). PCR reactions were conducted in a Roche quantitative PCR machine LC480 in a total reaction volume of 20 μl with SYBR Green PCR Master Mix (Takara, Kyoto, Japan). The PCR conditions were as follows: 50 °C for 2 min, 95 °C for 5 min, and 45 cycles of 95 °C for 40 s and 55 °C for 30 s. The primer sequences were as follows: β-actin sense: 5′-CTCCATCCTGGCCTCGCTGT-3′; β-actin anti-sense: 5′-GCTGTCACCTTCACCGTTCC-3′; UNC5B sense: 5′-CAGGGCAAGTTCTACGAGAT-3′; and UNC5B anti-sense: 5′-TGGTCCAGCAGGATGTGA-3′. The fold-change in UNC5B expression was normalized to β-actin and calculated using the ΔΔCT method. Experiments were performed in triplicate.

### Western blot analysis

Cells were washed with pre-cooled PBS 3 times, lysed in radioimmunoprecipitation assay (RIPA) buffer supplemented with phenylmethanesulfonyl fluoride protease inhibitor cocktail and centrifuged at 12,000 rpm for 30 min. Total proteins in the supernatant were collected. The protein concentration was determined using the BCA assay (Beyotime, Shanghai, China), and the values were normalized using a standard BSA curve. Then, 60 μg of standardized protein per lane was separated by electrophoresis in an SDS-PAGE gel and transferred onto polyvinylidene fluoride (PVDF) membranes. The membranes were incubated at 4 °C overnight with primary antibodies against UNC5B (1:1000) (Sigma, USA), GAPDH (1:2000) (Sigma, USA), cyclin B1 (1:1000) (Abcam, Hong Kong), cyclin D1 (1:1000) (Abcam, Hong Kong) and cyclin E (1:1000) (Abcam, Hong Kong). The membranes were subsequently incubated with secondary IgG antibody (Santa Cruz Biotechnology) at 37 °C for 1 h with shaking, and the bound proteins were visualized using the EC3 Imaging System (UVP Inc., Cambridge, UK).

### Immunofluorescence technique

Immunofluorescence analyses were conducted using 5637 and 5637-U cell lines cultured in 24-well plates. After the cells fusion, they were washed with PBS, permeabilized with 0.3 % Triton X-100 for 1 h, and 30 min at 37 °C of 5 % BSA. Then cells were incubated with UNC5B antibody (rabbit anti-human (1:1000)) overnight at 4 °C. After washing, the cells were incubated with TRITC-conjugated (labeled goat anti-rabbit IgG (1:200) secondary antibodies at 37 °C for 1 h in a dark place. Nuclear was stained with DAPI. Immunofluorescence images were observed utilizing an Inverted Flurescence Microscopy (Olympus, Tokyo, Japan).

### Cell cycle analysis

We cultured 5637 and 5637-U cells in serum-free medium for 12 h and subsequently cultured them in RPMI-1640 with 5 % FBS for an additional 24 h. Next, the cells were harvested, washed once with PBS, slowly combined with 75 % ice-cold ethanol and incubated overnight at 4 °C. The cells were then centrifuged at 1200 g, resuspended in PI/RNase Staining Buffer (Becton Dickinson Biosciences, San Jose, CA) and incubated for 30 min at 4 °C. The data of flow cytometry were analyzed using CellQuest Pro and ModFit software (Becton Dickinson Biosciences, San Jose, CA).

### Cell proliferation and wound healing assays

We used the Cell Counting Kit-8 (Beyotime, Shanghai, China) assay to compare the growth of 5637 and 5637-U cells. The two cell lines were plated at a density of 4.0 × 10^3^ cells per well in 96-well plates, and OD values were measured on each one of the 7 days. The experiments were performed according to the manufacturer’s protocol. 5637 and 5637-U cells were plated at a density of 1 × 10^5^ cells/well in 24-well plates and incubated in RPMI-1640 containing 10 % FBS for 24 h until they reached confluence. A wound was created in the adherent cells using a 200-μl pipette tip, followed by incubation with serum-free RPMI-1640 medium for 24 h. The changes in wound area were analyzed using an inverted microscope.

### In vivo mouse models of bladder cancer

Animal experiments were formally approved by the China Medical University Ethics Committee. Four-week-old female SPF/VAF nude mice weighing approximately 13 g were purchased from Vitalriver China. Stably transfected cells (5637-U) or normal cells (5637) (1 × 10^5^cells in 170 μl of RPMI1640 with 10 % FBS) were injected into the armpit or rear flank of nude mice to form implanted tumors. Tumor growth was monitored approximately every 2 days. At 47 days after injection, the mice were sacrificed, and the specimens (tumor or liver) were harvested for measurements and immunohistochemical analysis. The resected specimens were rinsed with PBS and fixed with 4 % formalin overnight.

### Immunohistochemistry staining

Fresh tissues harvested from mice were fixed with 4 % formalin for a minimum of 24 h. Next, the tissues were embedded in paraffin, sectioned and transferred to glass slides. Whole-mount immunostaining assays were conducted by incubating the slides with antibodies against UNC5B (1:200) (Sigma, USA), followed by secondary antibodies. The samples were then stained with DAB and rinsed. Nucleus were stained with hematoxylin (Beyotime, Shanghai, China) for 5 min and rinsed with water for more than 3 h. Images were captured using an Upright Metallurgical Microscope (Olympus, Tokyo, Japan).

### Statistical methods

We used the software SPSS for Windows 17.0 (SPSS Inc., Chicago, USA) for statistical analyses. Student’s *t*-test was used to analyze differences in UNC5B expression, cell migration and cell cycle arrest between 5637 and 5637-U cells. Analysis of variance of repeated measures was used to evaluate the growth curves of 5637 and 5637-U cells. *P* values <0.05 were considered statistically significant.

## Results

### Identification of stable clones

**Fig. 1:**
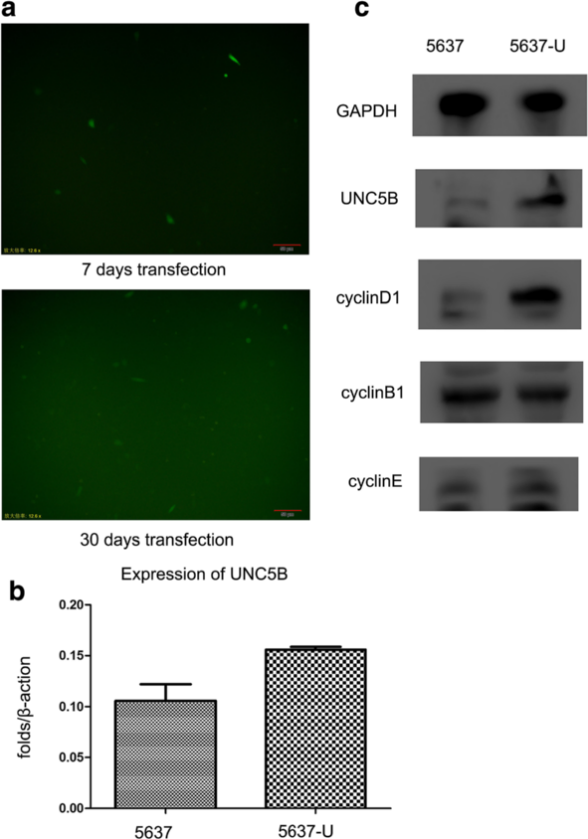
Transfection efficiency and expression of UNC5B in 5637 and 5637-U cells. **a** Detection of pcDNA-UNC5B-GFP in 5637-U cells using immunofluorescence 3 and 30 days after transfection. **b** Quantification of UNC5B expression levels in 5637 and 5637-U cells by real-time RT-PCR. **c** Evaluation of the stable transfection of 5637 cells with pcDNA-UNC5B-GFP by western blotting. Lane 1 represents untransfected 5637 cells, and lane 2 represents cells 2 weeks after transfection with pcDNA-UNC5B-GFP

As shown in Fig. 1a, the transfection efficiency of pcDNA-UNC5B-GFP in 5637 cells was investigated by fluorescence microscopy 7 days and 30 days after transfection and then selection with 500 μg/ml G418. After 30 days, a substantial number of 5637 cells exhibited green fluorescence, confirming stable transfection. The expression of UNC5B in 5637-U cells and control 5637 cells was evaluated by real-time RT-PCR and western blot assays. As shown in Fig. 1b, real-time PCR indicated that UNC5B expression was increased 12-fold in 5637-U cells compared with 5637 control cells (*P =* 0.037), and western blot analysis using GAPDH as a loading control revealed that UNC5B protein levels substantially increased in 5637-U cells compared with 5637 cells (Fig. 1c).

### UNC5B Overexpression decreases cell proliferation and migration

**Fig. 2:**
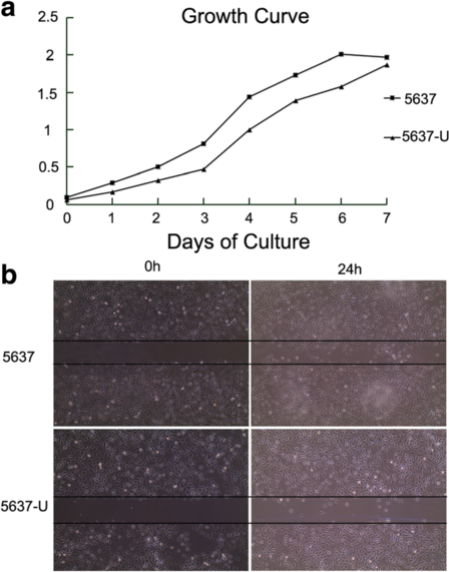
The proliferation and migration of 5637 cells is inhibited by UNC5B overexpression. **a** Evaluation of the proliferation of 5637 and 5637-U cells by the CCK-8 assay. The OD values of 5637 cells were higher than those of 5637-U cells on each of the 7 days examined. **b** Wound healing assays were used to examine cell migration in 5637 and 5637-U cells. Overexpression of UNC5B inhibited the migration of 5637 cells

For the CCK-8 experiment, we inoculated relatively few cells onto a 96-well plate and continuously observed the cells for 7 days. On all 7 days, the proliferation of 5637-U cells was reduced compared with 5637 cells (Fig. 2a). Significant differences were observed between days (*P <* 0.001), and the difference in the growth rate of 5637 and 5637-U cells was also significant (*P <* 0.001). Wound healing assays revealed significantly lower migration of 5637-U cells compared with 5637 cells (*P <* 0.05) (Fig. 2b).

### Location of UNC5B in 5637-U aggregates to cytoplasm

**Fig. 3:**
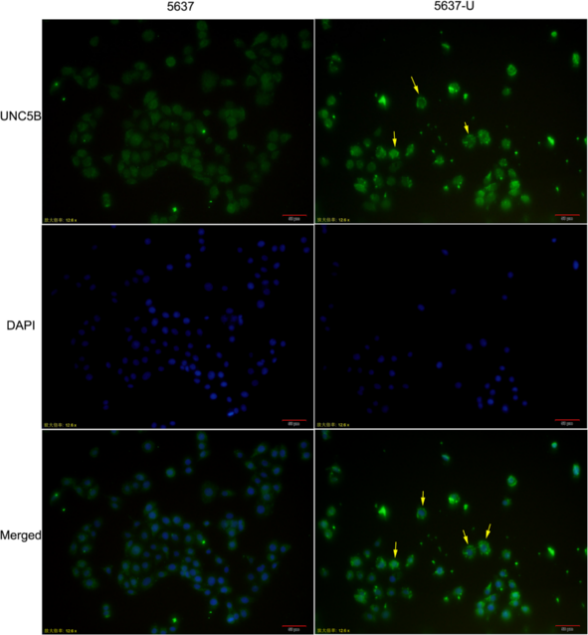
Location of UNC5B in 5637 and 5637-U cells assessed by immunofluorescence analyses. UNC5B (*green fluorescence*) was mainly expressed in cytoplasm while only a fraction located in nuclear. After UNC5B overexpressed, more *green* fluorescent spots assembled in cytoplasm as the *yellow* arrows showed

Immunofluorescence results indicated that UNC5B was mainly expressed in cytoplasm and a small number located in nuclear. After stably overexpressed UNC5B, more fluorescent spots aggregated in cytoplasm (Fig. 3).

### Overexpression of UNC5B Induces G2/M arrest in 5637-U cells

**Fig. 4:**
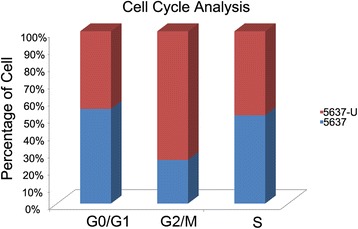
Flow cytometry analysis of cell cycle progression in 5637 and 5637-U cells. There was a significant increase in the number of 5637-U cells in G2/M phase compared with 5637 cells (15.34 % of 5637-U cells and 5.2 % of 5637 cells, *P =* 0.003)

Flow cytometry analysis indicated that cell cycle progression was significantly inhibited in 5637-U cells compared with 5637 cells (5637 cells: S phase 52.01 %; G2/M phase 5.2 %; G0/G1 phase 42.76 %. 5637-U cells: S phase 49.54 %; G2/M phase 15.34 %; G0/G1 phase 35.12 %) (*P =* 0.003) (Fig. 4). These data indicate that UNC5B overexpression inhibited proliferation and migration by inhibiting cell cycle progression at G2/M phase.

### The growth of tumors derived from 5637-U cells is reduced compared with 5637 cells

**Fig. 5:**
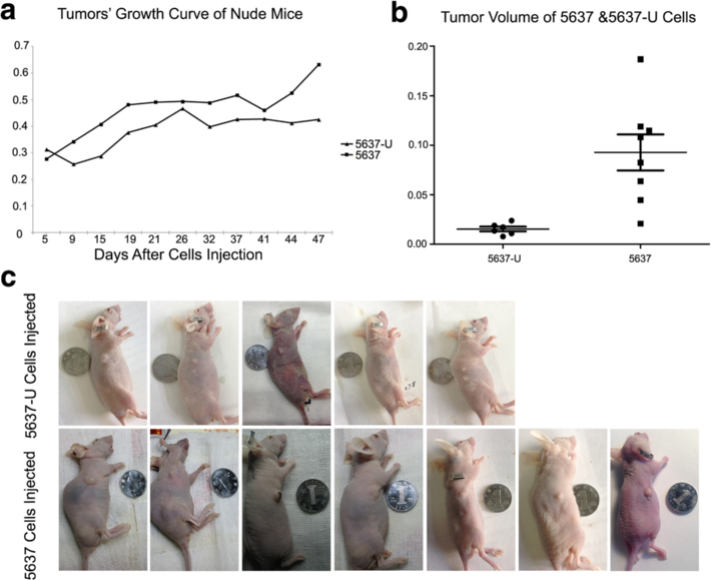
Tumors derived from UNC5B-overexpressing cells grew at a slower rate and were smaller in volume than tumors derived from 5637 cells. **a**, **b** The sizes of the tumors on the backs of the mice were recorded 5, 9, 15, 19 and 21…days after the injection of tumor cells. Tumors derived from 5637 cells grew at a faster rate and were larger in size than tumors derived from 5637-U cells on all days evaluated. **c** Representative images of the nude mice used in the tumor transplant experiments

Nude mice were injected with 5637 or 5637-U cells. The two cell lines resulted in the formation of tumors of different sizes on the back of mice and lethality at 32 days to 47 days after injection. UNC5D overexpression has been suggested to inhibit cell multiplication, migration and invasion in renal cancer cells by inducing cell cycle arrest at G2/M phase [12]. These data indicated that UNC5D may act as a tumor suppressor and are consistent with our findings in bladder cancer cells. We observed that tumors derived from 5637-U cells grew at a slower rate and were smaller than tumors derived from 5637 cells (*P =* 0.004) (Fig. 5a and b), implicating UNC5B as a candidate tumor suppressor in bladder cancer. Representative images of 12 nude mice, 5 injected with 5637-U cells and 7 mice injected with 5637 cells, are shown in Fig. 5c. In general, tumor formation requires the activation or inactivation of multiple signaling pathways, and in some cases, multiple inputs from related signaling pathways are required to induce tumor formation.

### Expression of cell cycle-associated proteins in 5637 and 5637-U cells

The expression of UNC5B and cell cycle proteinsin 5637 and 5637-U was evaluated by western blot analysis. The expression of cyclin D1 was enhanced in 5637-U cells compared with 5637 cells, whereas cyclin B1 and cyclin E levels were unaffected (Fig. 1c).

### Expression of UNC5B in 5637 and 5637-U-derived tumors

**Fig. 6:**
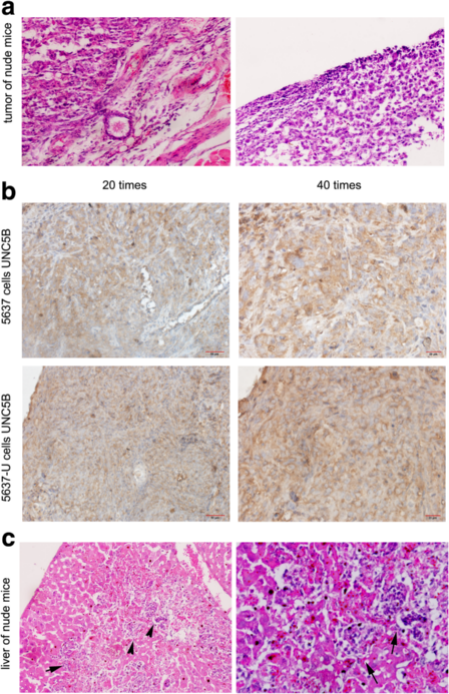
Representative images of tumor and liver tissues of nude mice evaluated by immunohistochemical staining. **a** H&E staining of the nude mouse tumors. **b** UNC5B (*brown staining*) localized to the cytoplasm and nuclear membrane in tumor tissues derived from 5637 and 5637-U cells. **c** Nude mice injected with 5637 cells exhibited liver metastases (*black arrows*)

Tumors and livers from the sacrificed mice were subjected to H&E and immunohistochemical analysis to determine the level of UNC5B expression (Fig. 6a). UNC5B protein expression was localized to both the cytoplasm and the nuclear membranes in the tumor tissues (Fig. 6b). Two of the mice injected with 5637 cells appeared moribund at 19 and 32 days, and dissection after sacrifice revealed hepatic metastasis (Fig. 6c).

## Discussion

Axon guidance factors and their cognate receptors function in processes beyond those associated with the central nervous system, including inflammatory and immunological responses, cancer cell growth, migration and apoptosis, and the response of the kidney to reperfusion injury [8, 13–15]. The UNC5B receptor is down-regulated in bladder cancer tissues and is associated with bladder cancer recurrence [10]. Therefore, we hypothesized that UNC5B signaling plays a key role in the development of bladder cancer. In this study, 5637 cells stably transfected with UNC5B (5637-U) exhibited a reduction in growth and wound healing ability compared with control 5637 cells. Consistent with these findings, UNC5B expression is enhanced in the non-aggressive bladder cancer cell lines BIU-87, and cell migration is decreased in BIU-87 cells overexpressing UNC5B [16]. The 5637-U cells grew at a slower rate, and tumors in nude mice derived from 5637-U cells were smaller than tumors derived from 5637 cells. Taken together, these data show that UNC5B may suppress the progression of bladder cancer. In addition, UNC5B overexpression induced cell cycle arrest at the G2/M phase andassembled more fluorescent spots in cytoplasm as the arrow pointed, thereby decreasing the proliferation and migration of 5637-U cells. Western blot analysis suggested that cyclin D1 expression was increased in 5637-U cells whereas cyclin B1 and cyclin E expression were unaffected, indicating that UNC5B may influence the proliferaton of bladder cancer cells by regulating the expression of proteins associated with cell cycle progression from G2/M phase. Moreover, hepatic metastases were identified in 2 nude mice injected with 5637 cells, whereas no metastases were observed in nude mice injected with 5637-U cells. Bruno Larrivée et al. demonstrated that UNC5B activation reduced angiogenesis [11], indicating that UNC5B overexpression may be a critical prognostic factor in bladder cancer metastasis. In summary, UNC5B is a potential tumor suppressor in bladder cancer; however, the precise mechanism by which UNC5B influences bladder cancer progression remains unclear and merits further investigation.

## Conclusion

Our results suggest that UNC5B overexpression inhibits the proliferation and migration of bladder cancer cells by inducing cell cycle arrest at G2/M phase.

## Abbreviations

CCK-8: Cell counting kit-8
PBS: Phosphate buffer saline
RT-PCR: Realtime-polymerase chain reaction
SDS-PAGE: Sodium dodecyl sulfate- polyacrylamide gel electrophoresis

## References

[1] Smaldone MC, Jacobs BL, Smaldone AM, Hrebinko RL (2008). Long-term results of selective partial cystectomy for invasive urothelial bladder carcinoma. Urology.

[2] Serafini T, Colamarino SA, Leonardo ED, Wang H, Beddington R, Skarnes WC, Tessier-Lavigne M (1996). Netrin-1 is required for commissural axon guidance in the developing vertebrate nervous system. Cell.

[3] Van Raay TJ, Foskett SM, Connors TD, Klinger KW, Landes GM, Burn TC (1997). The NTN2L gene encoding a novel human netrin maps to the autosomal dominant polycystic kidney disease region on chromosome 16p13.3. Genomics.

[4] Wang H, Copeland NG, Gilbert DJ, Jenkins NA, Tessier-Lavigne M (1999). Netrin-3, a mouse homolog of human NTN2L, is highly expressed in sensory ganglia and shows differential binding to netrin receptors. J Neurosci.

[5] Yin Y, Sanes JR, Miner JH (2000). Identification and expression of mouse netrin-4. Mech Dev.

[6] Shimizu A, Nakayama H, Wang P, Konig C, Akino T, Sandlund J (2013). Netrin-1 promotes glioblastoma cell invasiveness and angiogenesis by multiple pathways including activation of RhoA, cathepsin B, and cAMP-response element-binding protein. J Biol Chem.

[7] Delloye-Bourgeois C, Brambilla E, Coissieux MM, Guenebeaud C, Pedeux R, Firlej V, Cabon F, Brambilla C, Mehlen P, Bernet A (2009). Interference with netrin-1 and tumor cell death in Non–small cell lung cancer. J Natl Cancer Inst.

[8] Fitamant J, Guenebeaud C, Coissieux MM, Guix C, Treilleux I, Scoazec JY, Bachelot T, Bernet A, Mehlen P (2008). Netrin-1 expression confers a selective advantage for tumor cell survival in metastatic breast cancer. PNAS.

[9] Moore SW, Tessier-Lavigne M, Kennedy TE (2007). Netrins and their receptors. Adv Exp Med Biol.

[10] Liu J, Kong CZ, Gong DX, Zhang Z, Zhu YY (2014). PKC α regulates netrin-1/UNC5B-mediated survival pathway in bladder cancer. BMC Cancer.

[11] Larrivée B, Freitas C, Trombe M, Lv X, Delafarge B, Yuan L, Bouvrée K (2007). Activation of the UNC5B receptor by Netrin-1 inhibits sprouting angiogenesis. Genes Dev.

[12] Lu D, Dong D, Zhou Y, Lu M, Pang XW, Li Y (2013). The tumor-suppressive function of UNC5D and its repressed expression in renal cell carcinoma. Clin Cancer Res.

[13] Tadagavadi RK, Wang W, Ramesh G (2010). Netrin-1 regulates Th1/Th2/Th17 cytokine production and inflammation through UNC5B receptor and protects kidney against ischemia-reperfusion injury. J Immunol.

[14] Guenebeaud C, Goldschneider D, Castets M (2010). The dependence receptor UNC5H2/B triggers apoptosis via PP2A-mediated dephosphorylation of DAP kinase. Mol Cell.

[15] Wang W, Reeves WB, Ramesh G (2008). Netrin-1 and kidney injury. I. Netrin-1 protects against ischemia-reperfusion injury of the kidney. Am J Physiol Renal Physiol.

[16] Liu J, Zhang Z, Li ZH, Kong CZ (2013). Clinical significance of UNC5B expression in bladder cancer. Tumour Biol.

